# Epigenetic modification of histone 3 at lysine 9 in sheep zygotes and its relationship with DNA methylation

**DOI:** 10.1186/1471-213X-8-60

**Published:** 2008-05-29

**Authors:** Jian Hou, Lei Liu, Jing Zhang, Xiu-Hong Cui, Feng-Xiang Yan, Hong Guan, Yong-Fu Chen, Xiao-Rong An

**Affiliations:** 1 State Key Laboratory for Agrobiotechnology, College of Biological Science, China Agricultural University, Beijing 100094, PR China

## Abstract

**Background:**

Previous studies indicated that, unlike mouse zygotes, sheep zygotes lacked the paternal DNA demethylation event. Another epigenetic mark, histone modification, especially at lysine 9 of histone 3 (H3K9), has been suggested to be mechanically linked to DNA methylation. In mouse zygotes, the absence of methylated H3K9 from the paternal pronucleus has been thought to attribute to the paternal DNA demethylation.

**Results:**

By using the immunofluorescence staining approach, we show that, despite the difference in DNA methylation, modification of H3K9 is similar between the sheep and mouse zygotes. In both species, H3K9 is hyperacetylated or hypomethylated in paternal pronucleus relative to maternal pronucleus. In fact, sheep zygotes can also undergo paternal DNA demethylation, although to a less extent than the mouse. Further examinations of individual zygotes by double immunostaining revealed that, the paternal levels of DNA methylation were not closely associated with that of H3K9 acetylation or tri-methylation. Treatment of either 5-azacytidine or Trichostatin A did not induce a significant decrease of paternal DNA methylation levels.

**Conclusion:**

Our results suggest that in sheep lower DNA demethylation of paternal genomes is not due to the H3K9 modification and the methylated DNA sustaining in paternal pronucleus does not come from DNA *de novo *methylation.

## Background

During mammalian fertilization, two sets of genomes from male and female gametes join together and then undergo large-scale reprogramming to restore the totipotency. However, although reside in the same zygotic cytoplasm, the paternal and maternal genomes are reprogrammed in different ways. It is well known that several epigenetic modifications are involved in the reprogramming events [1,2].

The first described epigenetic modification is DNA methylation. DNA methylation at CpG dinucleotides is associated with the repression of gene transcription and is essential for mammalian development [3]. In mouse zygotes, the paternal genome undergoes active DNA demethylation shortly after fertilization, while the maternal genomic DNA remains methylated throughout the first mitosis [4,5]. Although the active demethylation of paternal genome has been observed in several mammalian species [6], with the same immunostaining approach no paternal DNA demethylation can be detected in sheep, rabbit and goat zygotes [7-9]. Therefore, the paternal demethylation event appears to be variable among species, but the mechanism underlying it is still unclear.

In addition to DNA methylation, covalent modifications of nucleosomal histone, including acetylation, methylation, phosphorylation and ubiquitination, also play critical roles in regulation of gene expression and are involved in the processes of epigenetic reprogramming [1,2]. Modifications can occur at several amino acid residuals, of which the lysine residue 9 of histone H3 (H3K9) can be either acetylated or methylated. In general, acetylated H3K9 represents gene transcription permissive status while methylated H3K9 mediates gene silencing [10]. In particular, H3K9 methylation has been suggested to be mechanistically linked to DNA methylation [11-13]. In mouse zygotes, methylated H3K9 is distributed asymmetrically between the maternal and paternal pronucleus [14-18], which is coincident with the distribution pattern of DNA methylation [4]. The absence of methylated H3K9 from the paternal pronucleus has been thought to attribute to the paternal DNA demethylation [17].

As sheep zygotes differ from mouse zygotes in the aspect of DNA methylation [7,9], it would be interesting to ask the question of whether the epigenetic differences are also reflected in histone modifications. To address this issue, this study detected the methylation and acetylation patterns of H3K9 in sheep zygotes and compared with that of the mouse zygotes. Furthermore, the possible relationship between H3K9 modification and DNA methylation was examined in sheep zygotes. Our results indicate that sheep zygotes display similar H3K9 modification patterns to the mouse and DNA methylation is not closely correlated with H3K9 modification.

## Results and Discussion

By immunostaining with specific antibodies, global histone modification patterns have been well characterized in mouse oocytes and zygotes. For convincible comparison purpose, we first established a satisfied staining protocol in mouse and then applied it to the detection in sheep. Each experiment was repeated at least 3 times and similar results were obtained.

### H3K9 acetylation patterns in oocytes and zygotes

**Figure 1:**
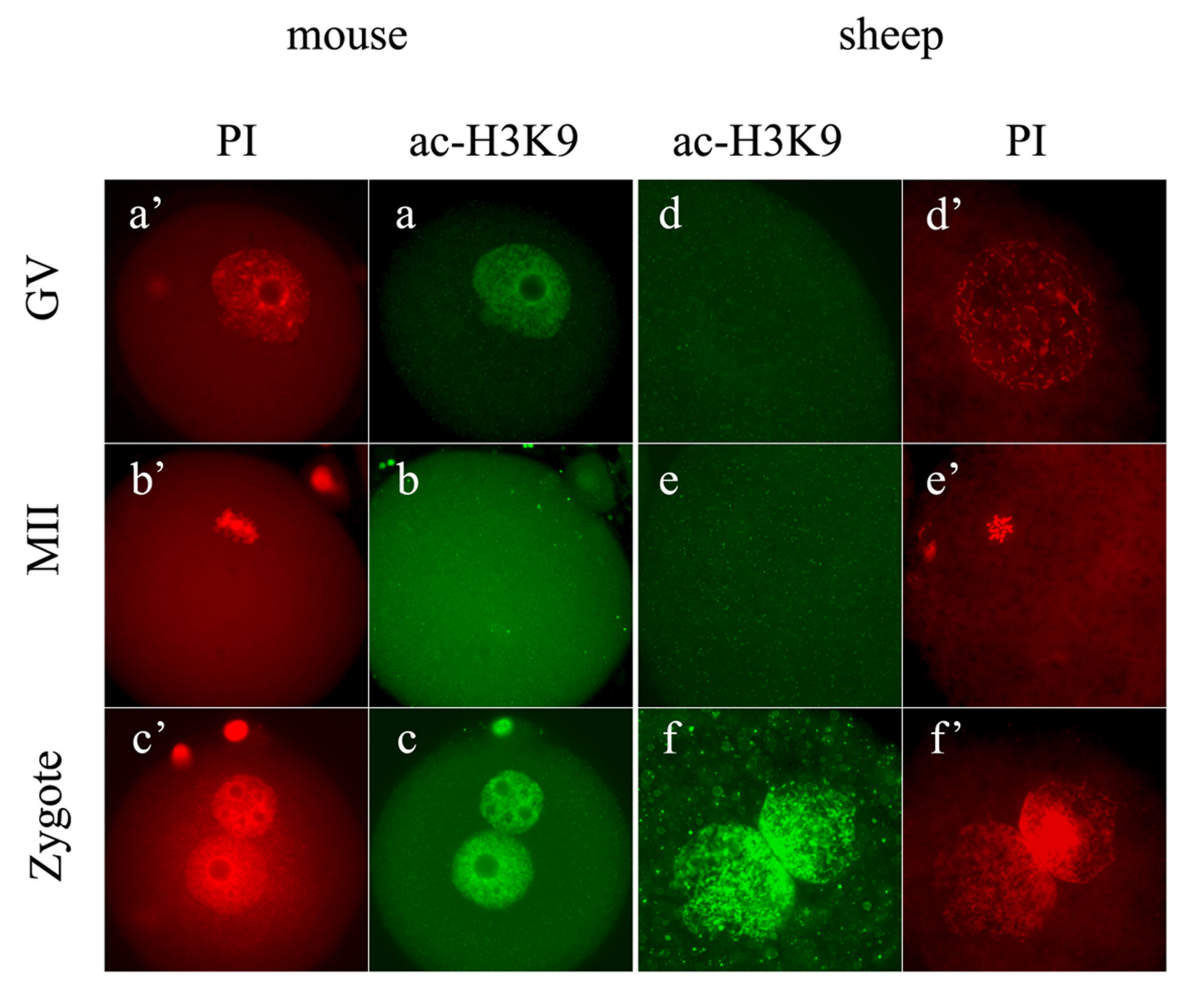
**H3K9 acetylation patterns in oocytes and zygotes**. In both mouse (a, a'-c, c') and sheep (d, d'-f, f'), GV-stage oocytes (a, d), MII-stage oocytes (b, e) and zygotes (c, f) were stained for H3K9 acetylation (ac-H3K9, green). The samples were counterstained with propidium iodide (PI, red) to visualize the DNA. Mouse GV nucleus showed positive staining for ac-H3K9 (a), but sheep GV nucleus showed no signal (d). In both species, MII-stage oocytes were negatively stained for ac-H3K9 (b and e). In zygotes (c and f), both parental pronuclei were intensively stained for ac-H3K9.

Our results from the detection of mouse oocytes and zygotes were consistent with that previously reported [17-19]. As shown in Figure 1, the nucleus of GV-stage oocytes was positively stained with ac-H3K9 antibody (Figure. 1a, a'), but the chromosomes of MII oocytes showed no staining for ac-H3K9 (Figure. 1b, b'). In the fully developed zygotes, both parental pronuclei were intensively labelled (Figure. 1c, c').

Unexpectedly to us, the acetylation of H3K9 could not be observed in the sheep GV nucleus (Figure 1d, d'). This observation was dramatically different from that in mouse (Figure 1a, a'). In mouse oocytes, histone acetylation disappears only after the occurrence of GV breakdown (GVBD) [19]. However, in this study, sheep oocytes showed no H3K9 acetylation from GV to MII stage (Figure 1d, e). Our results also conflict a recent report on sheep, where obvious signals for ac-H3K9 could be seen in the oocytes at all meiosis stages except MI [20]. It is not easy to give an explanation for this discrepancy. To confirm our result, we performed the same staining to the oocytes treated with TSA, a well-known deacetylase inhibitor. As expected, these oocytes showed a positive staining for ac-H3K9 (data not shown). Also, in the subsequent detection with sheep fertilized oocytes, the nucleus was heavily stained with ac-H3K9 antibody (see next). These results confirmed the specificity of the antibody used in our study. Based on our results, sheep oocytes seem to remain the deacetylation pattern of H3K9 during the course of in vitro maturation. Future studies may be needed to examine the acetylation state of other lysine residuals in sheep GV-stage oocytes.

Although no ac-H3K9 was detected in the oocytes, the zygotes displayed highly acetylated H3K9 in both parental pronuclei (Figure 1f, f'). This pattern was very similar to that in mouse (Figure 1c, c'). In mouse zygotes, acetylated histone is preferentially recruited by the forming paternal pronucleus and hyperacetylation in the paternal pronucleus relative to maternal pronucleus was maintained in pronuclear stage [21]. We performed quantitative analysis of the acetylation levels in the late zygotes and found that the paternal pronucleus was 13% (n = 17) more acetylated than the maternal pronucleus in mouse zygotes. In sheep zygotes, the slightly larger one of the two pronuclei was 21.7% (n = 23) more acetylated than its smaller counterpart. We have identified the larger ones as paternal pronuclei. This result suggests that the superiority of paternal hyperacetylation also exists in sheep zygotes.

### H3K9 tri-methylation patterns in oocytes and zygotes

In mouse oocytes, once global methylation patterns of H3K9 are established in fully grown oocytes [22], they remain constant during the period of oocyte maturation [23]. After fertilization methylated H3K9 is maintained in the maternal chromatin, but absent from the paternal chromatin throughout the first mitosis [14-18].

**Figure 2:**
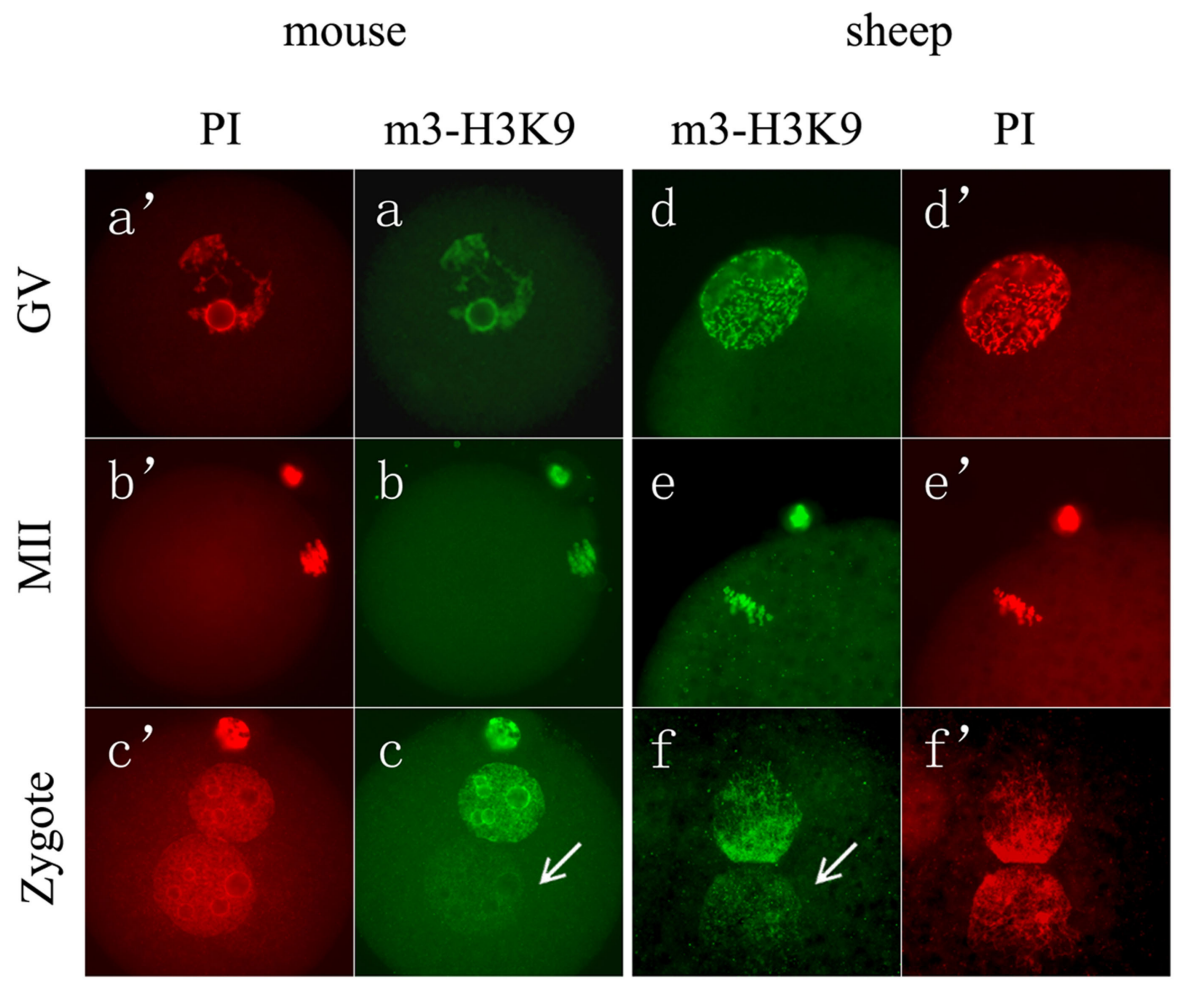
**H3K9 tri-methylation patterns in oocytes and zygotes**. In both mouse (a, a'-c, c') and sheep (d, d'-f, f'), GV-stage oocytes (a, d), MII-stage oocytes (b, e) and zygotes (c, f) were stained for H3K9 tri-methylation (m3-H3K9, green). The samples were counterstained with propidium iodide (PI, red) to visualize the DNA. In both species, the nucleus (a, d) or chromosomes (b, e) of oocytes were heavily stained for m3-H3K9. In zygotes (c and f), two parental pronuclei were asymmetrically stained for m3-H3K9, which the paternal pronucleus showed no or feint staining (arrow).

In this study, we showed that sheep oocytes displayed similar m3-H3K9 patterns to the mouse. As shown in Figure 2, in both species, m3-H3K9 was detected in both GV (Figure 2a, d) and MII oocytes (Figure 2b, e). In mouse zygotes, maternal pronucleus was associated with strongly stained m3-H3K9, but the paternal pronucleus was not (Figure 2c, c'). Similarly, in sheep zygotes, m3-H3K9 was observed only in one of the two pronuclei (n = 25/34) (Figure 2f, f'). The slightly larger one, showing no or faint signals of m3-H3K9, was supposed to be from the paternal origin. This was supported by the detection of parthenogenetic oocytes, in which the nucleus was intensively stained for m3-H3K9 as expected (data not shown). Quantitative analysis showed that the relative m3-H3K9 level of paternal pronucleus to maternal pronucleus was 57.8% (n = 22) in sheep zygotes, but 23.6% (n = 18) in mouse zygotes. The biological significance of this difference is unclear, but it may be due to the differences in the size of pronucleus and the degree of chromatin condensation between these two species. Compared to the mouse, sheep paternal pronucleus is less decondensed and is not much larger than its maternal counterpart. These factors might have some influences on the quantification of the paternal signal intensity relative to the maternal one.

We also noted in two recent articles from one research group that a *de novo *paternal H3K9 trimethylation event in several mammalian species was proposed except in the murine [24,25]. In pig and bovine zygotes, the paternal m3-H3K9 pattern was gradually established during pronucleus development. In our study, we did observe in some sheep zygotes (n = 7/34) that both pronuclei were positively stained for m3-H3K9, but we could not correlate these events with the zygote developmental stage. If sheep paternal pronucleus also underwent *de novo *H3K9 trimethylation, it would be expected that paternal H3K9 trimethylation was mostly seen in late zygote stage. However, we observed that even those zygotes at syngamy stage also exhibited different staining intensity of m3-H3K9 between two parental pronuclei (date not shown), implying that *de novo *H3K9 trimethylation did not occur in sheep zygotes. Future studies may be needed to elucidate the origin of the zygotes that both pronuclei were positively stained for m3-H3K9.

In general, sheep zygotes displayed m3-H3K9 asymmetry pattern between two parental pronuclei, as seen in the mouse. Taken together the results from H3K9 acetylation, these data indicate that in terms of H3K9 modification, sheep zygotes are largely similar to the mouse zygotes; that is, H3K9 is hyperacetylated or hypomethylated in paternal pronucleus relative to maternal pronucleus.

### DNA methylation patterns in zygotes

**Figure 3:**
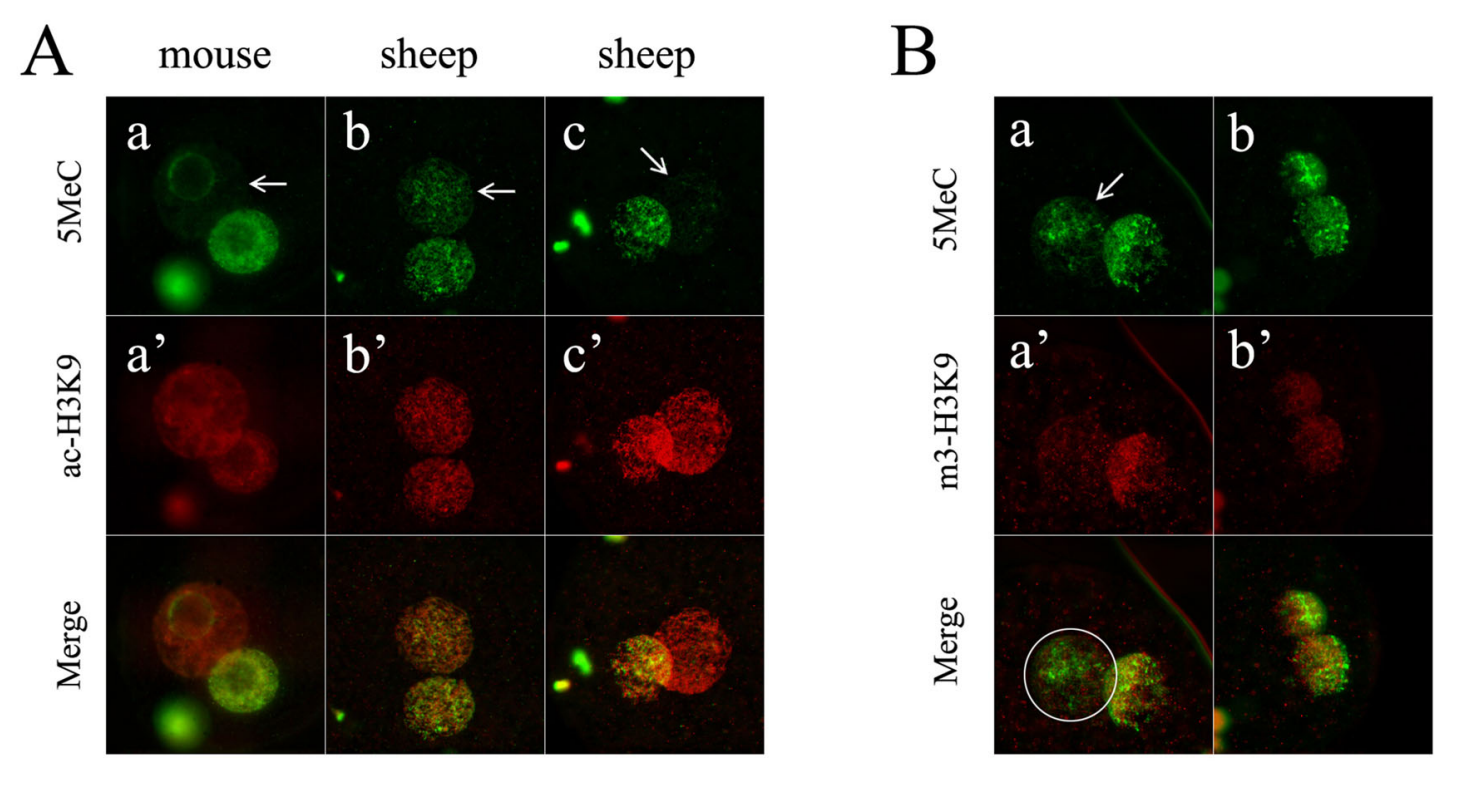
**DNA methylation patterns in zygotes and the relationship with H3K9 modification**. A. The zygotes were double stained for DNA methylation (5MeC, green) and H3K9 acetylation (ac-H3K9, red). In mouse zygote, no obvious signals of 5MeC could be seen in the whole paternal pronucleus except the perinucleolar regions (a, a'). In comparison, the paternal pronucleus in sheep zygote remained visible signals of 5MeC (b, b'). However, in some sheep zygotes, the paternal pronucleus showed very weak staining for 5MeC staining (c, c'). B. The zygotes were double stained for 5MeC (green) and H3K9 tri-methylation (m3-H3K9, red). In most cases, the paternal pronucleus remained some methylated regions (a), but m3-H3K9 was absent from the entire pronucleus area (a'). The boundary of paternal pronucleus is indicated by a white circle in the merged image. In b and b', both 5MeC and m3-H3K9 distributed equally in two parental pronuclei. This situation occurred in a few cases. All arrows in images were used to indicate the paternal pronucleus.

It has been well demonstrated in mouse that paternal genomic DNA is rapidly and actively demethylated soon after fertilization [4,5]. Although the active demethylation of paternal genomic DNA has been observed in several mammalian species [6], it appears not to be conserved for all mammals [7]. Results of previous studies of us and of others showed that sheep zygotes lacked paternal-specific demethylation, in marked contrast to the mouse [7,9]. In the present study, we re-examined this modification and quantitatively analyzed the immunofluorescence intensities. We have modified the immunostaining procedure (the 5MeC antibody concentration was highly diluted and the staining with primary antibody was carried out by overnight incubation at 4°C, see Methods). As shown in Figure 3A, DNA methylation state almost completely lost in mouse paternal pronucleus (Figure 3A. a, a'), but was preserved in sheep paternal pronucleus (Figure 3A. b, b'). Clearly, by simple visual assessment, sheep zygotes did not undergo a dramatic paternal demethylation, which is in consistent with previous description [7,9]. However, quantitative analysis revealed that the average DNA methylation level in the paternal pronucleus was approximately 30% less than that in the maternal counterpart (n = 22). This difference was significant, thus reflecting that paternal demethylation dose occur to some extent in sheep zygotes. However, we noted that sheep zygotes showed more heterogeneous paternal demethylation than mouse zygotes. Most strikingly, in 23% (5/22) of the sheep zygotes tested, the paternal pronucleus almost lost all of their methylation, which resulted in an obvious asymmetric 5MeC staining pattern between the two parental pronuclei (Figure 3A. c, c'). In these zygotes, the relative DNA methylation levels of paternal pronucleus to maternal pronucleus declined below 50%. This observation provides a cue that sheep zygotes could undergo genome-wide demethylation of the paternal genome, under some unknown circumstances. Previous studies demonstrated that sheep oocyte cytoplasm does have the capability of demethylating the injected mouse sperm or transferred sheep somatic cell nucleus [26,27]. Our present findings add new evidences supporting the claim that sheep oocytes contain considerable demethylation activity, although less than the mouse oocytes [26].

### Relationship between H3-K9 modification and DNA methylation

The association of DNA methylation with histone modification has been suggested in other systems [11-13,28] and mouse zygotes [17]. We tested this association in sheep zygotes. Double immunostaining with their relevant antibodies was performed to co-locate these two epigenetic events. The results showed that the paternal pronucleus with hypomethylated DNA was generally coupled with hyperacetylated (n = 13/18) (Figure 3A) or undermethylated H3K9 (n = 11/17) (Figure 3B). However, we could not establish a close correlation between DNA methylation and H3K9 modification. When individual embryos were analyzed, we found that the degree of paternal DNA demethylation was not strictly correlated with that of H3K9 hyperacetylation.

It is thought that the presence of methylated H3K9 in the maternal pronucleus protects the maternal genomic DNA from active demethylation, while the absence of H3K9 methylation in paternal pronucleus allows active DNA demethylation to occur [17]. In mouse zygotes produced by fertilization with spermatides, the demethylation-resistant regions in paternal pronucleus were overlapped with m3-H3K9 [29]. These studies suggest a connection between DNA methylation and H3K9 methylation. However in our detection, in a majority of sheep zygotes tested (11/17), 5MeC signals were retained on some regions of the paternal pronucleus, while m3-H3K9 was totally absent from the paternal pronucleus (Figure 3B. a, a'). This result indicates that in sheep zygotes the maintenance of methylated DNA in paternal pronucleus was not absolutely dependent on the presence of methylated H3K9. However, we also observed in some cases (4/17) that m3-H3K9, along with methylated DNA, equally distributed in both pronuclei (Figure 3B. b, b'). As no paternal DNA demethylation appeared to happen in these zygotes, it raised the question of whether or how H3K9 methylation exerted influences on this situation. Recent studies claimed that H3K9 tri-methylation as well as di-methylation did occur in paternal pronucleus in pig and bovine zygotes [24,25,30]. In contrast to previous reports [6,7], those studies found that pig paternal pronucleus showed no genome-wide DNA demethylation, while bovine paternal pronucleus underwent an initial DNA demethylation followed by immediate remethylation. In either case of pig or bovine, the authors believed that DNA methylation event was closely associated with H3K9 tri-methylation [24,25]. In our study, m3-H3K9 was absent from the paternal pronucleus in most sheep zygotes even those at syngamy stage, meanwhile 5MeC was preserved in paternal pronucleus, although at various levels among individual embryos. According to these observations, the lower demethylation in sheep paternal pronucleus is not due to the H3K9 methylation.

### Effects of 5-aza and TSA treatments on paternal demethylation

**Figure 4:**
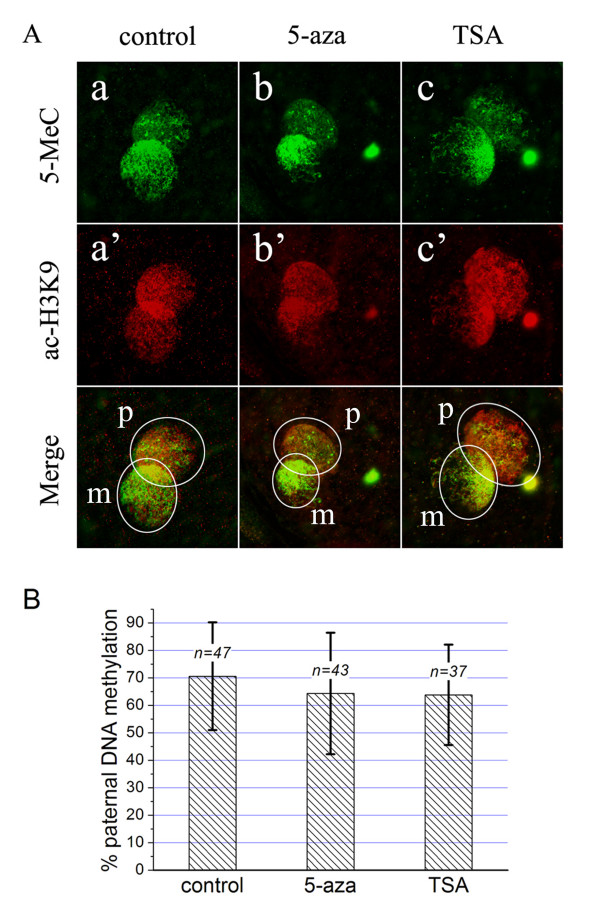
**Effects of 5-azacytidine or Trichostatin A on paternal demethylation**. A. The oocytes were treated with 5-azacytidine (5-aza) or Trichostatin A (TSA) during the period of fertilization, and then they were double stained for DNA methylation (5MeC, green) and H3K9 acetylation (ac-H3K9, red). When compared to the untreated zygotes (control, a), the zygotes treated with 5-aza (b) or TSA (c) showed no significant changes in paternal DNA methylation levels. However, TSA treatment increased the immunoflurescence for ac-H3K9 (a', b', c'). In the merged images, the pronucleus area is marked by a white circle (p: paternal, m: maternal). B. Quantitative analysis of the DNA methylation levels in paternal pronucleus. The percentage of paternal DNA methylation indicates the DNA methylation levels in paternal pronucleus relative to maternal pronucleus (mean ± SD). The number of zygotes analyzed is indicated as "*n*=" above each column. Student's *t *test revealed no significant differences among groups (p > 0.05).

Previous study has shown that in spermatide-fertilized mouse zygotes, the paternal DNA remethylation could be prevented by DNA methyltransferase inhibitor 5-aza or histone deacetylase inhibitor TSA [29]. Similar situation was also observed in bovine zygotes, which initial demethylation of paternal DNA was followed by remethylation and 5-aza treatment counteracted this process [25]. To investigate whether the methylated DNA sustaining in sheep paternal pronucleus virtually comes from remethylation event, we treated the oocytes with 5-aza or TSA during fertilization. The results showed that treatment with these drugs did not induce a significant decrease of paternal DNA methylation (Figure 4A and 4B). As that observed in untreated zygotes (control), the paternal DNA methylation levels in 5-aza or TSA treated zygotes also varied among individuals. Genome-wide loss of paternal 5MeC signals only occurred in 28% and 30%, respectively, of the zygotes from 5-aza and TSA treatment group, not significantly different from 25% of control group. Based on the average levels of paternal DNA methylation, there were no significant differences among three groups (Figure 4B). These results suggest that the observed 5MeC signals in sheep paternal pronucleus might not be from DNA remethylation, but probably from incomplete or partial demethylation.

The mechanism of the incomplete demethylation in sheep paternal genome is unclear. One consideration is the structure of the genome organization. Generally, sheep pronucleus is less decondensed than mouse pronucleus. It appears that some regions, if not all, of sheep sperm genome are inherently refractory to the demethylation process. Even injected into the mouse oocyte cytoplasm that contains higher demethylation activity, these regions, probably with compact organization, retain some methylation [26].

In addition, TSA treatment indeed increased the acetylation levels, but 5-aza treatment did not (Figure 4A, a'–c'). Both 5-aza and TSA had no effects on H3-K9 tri-methylation (data not shown), in agreement with previous report [25]. The involvement of related enzymes, for example DNA methyltransferase, in regulation of the methylation pattern of pronucleus-stage zygotes, is seldom known. It was shown in one more recent report, that DNA methyltransferase 1s (DNMT1s) is differentially localised between the paternal and maternal pronuclei of mouse zygotes, implying that this enzyme may attribute to the asymmetric DNA methylation between parental genome [31]. If it is the case, future studies may be expected to investigate the role of DNMT1 in maintenance of DNA methylation in sheep zygotes.

## Conclusion

In the present study we have shown that sheep zygotes displayed similar H3K9 modification patterns to the mouse, and sheep zygotes could also undergo paternal DNA demethylation, although to a less extent than the mouse. We have further indicated that the lower DNA demethylation in sheep paternal pronucleus is not due to the H3K9 modification, and the methylated DNA retaining in sheep paternal pronucleus does not arise from DNA remethylation. Our data presented here may help to further understand the epigenetic differences among mammalian species.

## Methods

### Collection of the oocytes and zygotes

All the mouse samples were derived from 4 to 5 week-old female Kun Ming strain mice. Immature and mature oocytes were collected respectively from the follicles and oviducts of superovulated female mice. Mouse zygotes were obtained from superovulated females as previously described [9].

Sheep oocytes were derived from the abattoir-collected ovaries and the zygotes were produced by oocyte *in vitro *maturation (IVM) and *in vitro *fertilization (IVF). The oocytes were recovered by aspiration of follicles 2 to 5 mm in diameter, and the oocytes with evenly granulated cytoplasm and enclosed by more than 3 layers of cumulus cells were selected for IVM. The IVM medium was TCM199 (Sigma-Aldrisch, St Louis, MO, USA) supplemented with 20% estrual sheep serum (eSS, made by this lab), 10 μg/ml FSH (Vetrepharm, Bioniche, Canada), 10 μg/ml LH (Lutropin, Bioniche, Canada) and 1 μg/ml 17β-estradiol (Sigma-Aldrisch). The oocytes were matured for 20 to 22 h at 38.5°C in 5% CO_2 _in humidified air. Germinal vesicle (GV) stage oocytes were harvested immediately after aspiration of the follicles and metaphase II (MII ) stage oocytes were collected from 20 to 22 h after the onset of oocyte maturation.

IVF was carried out according to the procedure described previously [9]. Briefly, frozen-thawed sperm were directly layered under 600 μl of fertiliztion medium and incubated for 30 min at 38.5°C for swimming up. Fertilization medium consisted of synthetic oviducal fluid medium (SOF) supplemented with 2% (v/v) eSS. The matured oocytes were placed into the four-well dishes with each well containing 450 μl fertilization medium covered with mineral oil, and then 50 μl of the swim-up fraction of spermatozoa were added into the wells, giving a final concentration of 1 × 10^6 ^spermatozoa/ml. After sperm-oocytes were co-incubated for 18–20 h in the fertilization medium, they were collected for immuofluorescence staining. For the 5-azacytidine (5-aza, Sigma-Aldrisch) or Trichostatin A (TSA, Sigma-Aldrisch) treatment of fertilized eggs, a final concentration of 10 μM 5-aza or 1 μM TSA was added into the fertilization medium.

### Immunofluorescence Staining

After collection, the oocyte or embryo samples were washed twice in PBS and fixed overnight at 4°C in 4% paraformaldehyde. Fixed samples were washed twice in PBS containing 0.05% Tween-20, and permeabilised with 0.5% Triton X-100 for 45 min at room temperature. The samples were blocked overnight at 4°C in 5% goat serum, 2% BSA and 0.1% Triton X-100 in PBS. To detect the patterns of H3K9 methylation or acetylation, the samples were incubated overnight at 4°C with the appropriate primary antibodies. After three 15-min washes, the samples were incubated in a FITC conjugated anti-rabbit secondary antibody (Sigma-Aldrisch, 1:200) for 1 h at 37°C.

The primary antibodies used here were rabbit polyclonal anti-trimethylated K9 (m3-H3K9, 1:200) or anti-acetylated K9 (ac-H3K9, 1:400) of histone H3 antibodies (Upstate Biotechnology, Lake Placid, NY). If the treatments were for the detection of DNA methylation, after permeabilisation the samples were treated with 2 N HCl for 1 h, then extensively washed with PBS/0.05% Tween-20 and blocked as described above. To detect the DNA methylation patterns, a mouse monoclonal anti-5methylcytosine (5MeC) antibody (Eurogentec, Seraing, Belgium, 1:800) was used as primary antibody. Primary antibody was detected with a FITC conjugated anti-mouse secondary antibody (Sigma-Aldrisch, 1:200). Samples were counterstained with propidium iodide (PI, Sigma-Aldrisch) to visualize the DNA, and then mounted onto the slides in Dabco antifade solution for observations.

Double immunostaining of DNA methylation and histone modification was accomplished by sequential incubation of the relevant primary and secondary antibodies. FITC-conjugated anti-mouse (Sigma-Aldrisch) or Rhodamine-conjugated anti-rabbit secondary antibodies (SouthernBiotech, Birmingham, AL) were used to detect anti-5MeC or anti-H3K9 primary antibodies, respectively.

Observations were performed with an Olympus BX51 epifluorescence microscope with excitation wavelengths of 501 (for FITC) and 568 nm (for PI or Rhodamine). All images were recorded digitally with a high-resolution CCD camera.

### Quantitative Analysis

The integrated fluorescence intensities of parental pronuclei were measured using ImageJ software. After subtracting the background of the images, each pronucleus was manually outlined and exposed to measure for quantifying the fluorescence intensities. The relative fluorescence intensities of paternal pronucleus to maternal pronucleus were calculated and indicated as the percentage (%) of paternal versus maternal intensity. Student's *t *test was used to compare the data obtained from the groups with 5-aza, TSA treatment and control and difference at P < 0.05 was considered significant.

## Authors' contributions

JH designed the experiment, performed most experiment work and drafted the manuscript. LL contributed to the production of sheep embryos. JZ and X–HC prepared the mouse embryos and participated in the immunostaining procedure. F–XY and HG helped to prepare the experiment materials. Y–FC provided critical review for the results and manuscript content. X–RA supervised and coordinated the study. All authors read and approved the final manuscript.
